# The X-Rays in the Discovery of Gall-Stones

**Published:** 1898-05

**Authors:** 


### THE X-RAYS IN THE DISCOVERY OF GALL-STONES.

Buxbaum (Carlsbad) recommends the use of photography in searching for gall-stones. The patient is placed upon his face with the photographic plate under the abdomen and the tube just over him. In this way clear-cut pictures of the gall-stones in the gall-bladder may be obtained. The possibility of making these discoveries is of great value in medicine and opens up another field of usefulness for the X-rays.—*Revue med de therapeut.*, No. 2, 1898.

